# Anti‐BCMA Immuno‐NanoPET Radiotracers for Improved Detection of Multiple Myeloma

**DOI:** 10.1002/adhm.202101565

**Published:** 2021-11-07

**Authors:** Eloise Thomas, Clélia Mathieu, Patricia Moreno‐Gaona, Vincent Mittelheisser, François Lux, Olivier Tillement, Xavier Pivot, Paiman Peter Ghoroghchian, Alexandre Detappe

**Affiliations:** ^1^ LAGEPP Université Claude Bernard Lyon 1 CNRS UMR5007 Villeurbanne France; ^2^ Université Paris‐Saclay CNRS UMR 8612 Institut Galien Paris‐Saclay France; ^3^ Institut de Cancérologie Strasbourg Europe (ICANS) Strasbourg France; ^4^ Institut Lumière‐Matière Université Claude Bernard Lyon 1 CNRS UMR5306 Villeurbanne France; ^5^ Institut Universitaire de France (IUF) Paris France; ^6^ David H Koch Institute for Integrative Cancer Research MIT Cambridge MA USA; ^7^ Dana Farber Cancer Institute Boston MA USA; ^8^ Strasbourg Drug Discovery and Development Institute (IMS) Strasbourg France

**Keywords:** diagnostics, multiple myeloma, positron emission tomography, targeted‐nanoparticles

## Abstract

Current clinical imaging modalities for the sensitive and specific detection of multiple myeloma (MM) rely on nonspecific imaging contrast agents based on gadolinium chelates for magnetic resonance imaging (MRI) or for ^18^F‐FDG‐directed and combined positron emission tomography (PET) and computed tomography (CT) scans. These tracers are not, however, able to detect minute plasma cell populations in the tumor niche, leading to false negative results. Here, a novel PET‐based anti‐BCMA nanoplatform labeled with ^64^Cu is developed to improve the monitoring of these cells in both the spine and femur and to compare its sensitivity and specificity to more conventional immunoPET (^64^Cu labeled anti‐BCMA antibody) and passively targeted PET radiotracers (^64^CuCl_2_ and ^18^F‐FDG). This proof‐of‐concept preclinical study confirmed that by conjugating up to four times more radioisotopes per antibody with the immuno‐nanoPET platform, an improvement in the sensitivity and in the specificity of PET to detect tumor cells in an orthotopic model of MM is observed when compared to the traditional immunoPET approach. It is anticipated that when combined with tumor biopsy, this immuno‐nanoPET platform may improve the management of patients with MM.

## Graphical abstract

Current immunoPET radiotracers are limited to 2–4 radioisotopes per monoclonal antibody (mAb). Here, a novel immuno‐nanoPET radiotracer is developed where the mAb is conjugated to three nanoparticles, each presenting four radioisotopes. When conjugated to an anti‐BCMA mAb, this construct enables improved sensitivity and specificity than convention immunoPET approach for multiple myeloma monitoring.

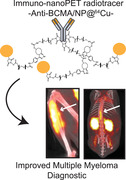

## 1 Introduction

Imaging modalities for the early detection and longitudinal tracking of multiple myeloma (MM) have been significantly improved due to the optimization of novel magnetic resonance imaging (MRI) acquisition protocols and positron emission tomography (PET) tracers.^[^ ^1^, ^2^, ^3^, ^4^ ^]^ While MRI and PET tracers are routinely used to diagnose and monitor the progression of MM, these tracers are far from optimal due to their non‐specific binding to tumor cells. MRI tracers accumulate in tumor environments based on the enhanced permeability and retention (EPR) effect,^[^ ^5^ ^]^ while PET radiotracers, and more specifically the ^18^F‐FDG tracer, function by imaging the tumor based on its glucose uptake, which, at low density, remains nonspecific^[^ ^6^, ^7^ ^]^ and can lead to false negative results in the context of cellular inactivity.^[^ ^8^ ^]^

Recently, immunoPET radiotracers have been translated into the clinic due to the specificity of selected antibodies and the high sensitivity of PET imaging^[^ ^9^, ^10^ ^]^. Preclinical studies involving immunoPET radiotracers (using antibodies or nanobodies)^[^ ^11^, ^12^, ^13^ ^]^ and antibody‐conjugated MRI tracers^[^ ^14^ ^]^ have demonstrated the possibility of significantly improving longitudinal monitoring of MM. The development of novel immunoPET radiotracers is of keen interest due to their better signal‐to‐noise ratios (SNRs) over MRI‐based methods and their ability to detect small clusters of cells, potentially enabling the identification of minimal residual disease (MRD), which is currently difficult with passive contrast agents for PET imaging.^[^ ^3^ ^]^ Importantly, recent preclinical and clinical studies have demonstrated promising results with the development of novel immunoPET radiotracers based on the use of anti‐CD38 (mAb) and anti‐CD138 monoclonal antibodies (mAbs) conjugated with DOTA‐^64^Cu or DOTA‐^89^Zr to monitor MM cells.^[^ ^11^, ^12^ ^]^ The results of the first clinical trials in MM patients using these tracers^[^ ^12^ ^]^ demonstrated that the use of CD38‐immunoPET radiotracers improved both the sensitivity and specificity of MM detection in comparison to fluorodeoxyglucose (^18^F) (^18^F‐FDG)‐based PET imaging.

Leveraging these promising results, we designed an innovative approach that targets the B‐cell maturation antigen (BCMA) that is widely used as a therapeutic target in MM and that is highly expressed at all stages of the disease; notably, this approach differs from traditional methods that have instead targeted CD38, which is a transmembrane glycoprotein that is almost exclusively expressed on MM cells.^[^ ^14^ ^]^ BCMA is a known target for chimeric antigen receptor (CAR) T‐cell^[^ ^15^ ^]^ and bispecific antibody therapy;^[^ ^16^ ^]^ but, it has yet to be adopted for MM imaging.^[^ ^17^ ^]^ While we previously successfully demonstrated that targeting BCMA for preclinical MRI‐based imaging of MM is a relevant approach,^[^ ^14^ ^]^ here, we hypothesized that the use of a immuno‐nanoPET radiotracer would enable a decrease in the amount of mAb needed for injection in comparison to the amounts of immunoPET radiotracer required to improve the sensitivity of PET imaging. To develop this platform, we conjugated polysiloxane‐based ultrasmall (<5 nm) nanoparticles (NPs) with NODAGA chelators for efficient ^64^Cu labeling. Then, further functionalization between the NP‐NODAGA and the anti‐BCMA mAb was performed to generate the anti‐BCMA/NP conjugate. We selected the radioisotope ^64^Cu instead of ^89^Zr because of its shorter half‐life (12.7 h vs. 3.3 days, respectively), enabling longitudinal injections in a clinically relevant workflow.

The functionalization of the polysiloxane matrix to NODAGA chelators was performed on a polysiloxane matrix presenting already chelated gadolinium atoms in DOTAGA chelates.^[^ ^18^, ^19^ ^]^ This grafting approach is similar to the previously described protocol performed to conjugate a second metal to the surface of the same NP.^[^ ^20^ ^]^ Additionally, we previously described that such NPs are safe and nontoxic after conjugation to various antibodies, including an anti‐BCMA mAb.^[^ ^14^, ^21^ ^]^ However, we did not use the intrinsic gadolinium MRI properties for bimodal imaging in this study as we focused only on evaluating PET imaging sensitivity and specificity in comparison to conventional PET contrast agents

**Figure 1:**
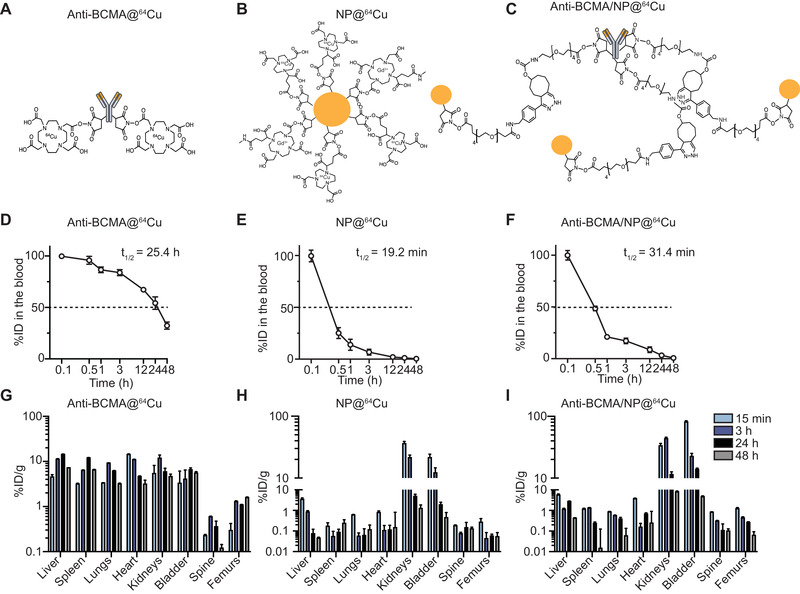
Design and pharmacokinetic profiles of the radioisotopes. A) Structures of the anti‐BCMA@^64^Cu immunoPET, B) untargeted NP@^64^Cu, and C) anti‐BCMA/NP@^64^Cu immuno‐nanoPET radiotracers. D) Pharmacokinetic profiles of anti‐BCMA@^64^Cu, E) NP@^64^Cu, and F) anti‐BCMA/NP@^64^Cu in healthy BALB/c mice (*n* = 5/time point). **G**. Biodistribution data in the major organs over time in anti‐BCMA@^64^Cu, H. NP@^64^Cu, and I) anti‐BCMA/NP@^64^Cu healthy BALB/c mice (*n* = 5/time point). All measurements were decay‐corrected.

The motivation to functionalize anti‐BCMA mAbs with NPs was based on two reasons: i) we previously demonstrated that the anti‐BCMA mAb could be loaded with up to 4 NPs on its structure without affecting its targeting specificity in vitro or in vivo and that this conjugation effectively reduced the half‐life and improved the clearance of the mAb from the body;^[^ ^14^ ^]^ ii) while the synthesis of the currently used immunoPET tracers is limited to approximately 2 to 4 radioisotopes per antibody,^[^ ^11^, ^12^ ^]^ immuno‐nanoPET tracers allow optimization of the number of molecules per mAb (i.e., the drug‐to‐antibody ratio (DAR)) up to of 4^[^ ^14^ ^]^ and enable an increase amount of radiotracer per mAb (as each NP can carry several radionuclides).^[^ ^22^ ^]^ In this study, we functionalized an anti‐BCMA mAb with ultrasmall NPs bearing a NODAGA chelator filled with the radioisotope ^64^Cu (i.e., anti‐BCMA/NP@^64^Cu). We compared its sensitivity and specificity to detect MM cells in an orthotopic MM mouse model and to results obtained with anti‐BCMA immunoPET (anti‐BCMA@^64^Cu), untargeted NP@^64^Cu, ^64^CuCl_2_, and ^18^F‐FDG radiotracers (**Figure** **1**A–C).

## 2 Results and Discussion

The synthesis of the immune‐nanoPET radiotracer was performed in three steps. First, ultrasmall NPs of approximately 3.6 nm in diameter were synthesized; their surfaces were functionalized with NODAGA chelators, leading to small particles with diameters of 4.3 nm.^[^ ^22^ ^]^ The addition of approximately 4 NODAGA chelators to the particle surface was confirmed with several complementary techniques, including zetametry, infrared spectroscopy, and titrations with europium and copper ions (see Materials and Methods and Figure S1, Supporting Information). In the second step, NP/NODAGA was grafted onto the mAb, using a previously reported transcyclooctene (TCO)‐tetrazin (Tz) click chemistry approach^[^ ^21^ ^]^ and leading to approximately 3 NPs per antibody. Finally, the anti‐BCMA/NPs were successfully labeled with ^64^Cu and purified to give 100% radiochemical purity. In comparison to classical immunoPET tracers, the NP‐based immunoPET strategy has an advantage in its ease of loading for an increased number of radioisotopes per mAb; and, with up to 3 NPs per antibody and 4 NODAGA chelators per NP, we were able to conjugate up to 12 radioisotopes per mAb instead of the typical 2 to 4 radioisotopes obtained through standard immunoPET approaches. Importantly, the conjugation of an average of 3 NPs per anti‐BCMA mAb does not interfere with its binding affinity (Figure S2, Supporting Information).

An in vivo evaluation of the developed immuno‐nanoPET tracer was then performed on an orthotopic xenograft MM mouse model. The conjugation of the ultrasmall NPs to monoclonal antibodies (mAbs) significantly decreased the circulation time of the mAbs (31.4 min for anti‐BCMA/NP@^64^Cu vs. 25.4 h for anti‐BCMA@^64^Cu) in comparison to the anti‐BCMA@^64^Cu immunoPET tracer (Figure 1D–F). The pharmacokinetic (PK) results showed significantly different biodistribution profiles for anti‐BCMA@^64^Cu in comparison to NP@^64^Cu and anti‐BCMA/NP@^64^Cu (Figure 1G–I). While anti‐BCMA@^64^Cu seems to be equivalently represented in the majority of organs from 15 min to 48 h after intravenous (IV) administration in healthy mice, rapid washout through the kidneys was observed for both the untargeted and targeted NP platforms, which was in line with results from previous studies.^[^ ^14^ ^]^

A comparative study was performed by determining the optimal SNR based on the diverging BD profiles of the biomarkers (Figure 1A). No signs of macroscopic toxicity were observed after IV injection of the different tracers as evidenced from stable body weights during monitoring (Figure S3, Supporting Information) and on previously conducted in‐depth blood and tissue analyses after hematoxylin and eosin staining.^[^ ^14^ ^]^ The biodistribution profile of anti‐BCMA/NP@^64^Cu is similar to that from the previously described anti‐BCMA/NP@Gd^3+^ compound.^[^ ^14^ ^]^ We first validated the specificity of the anti‐BCMA/NP@^64^Cu to target BCMA+ cells in vivo by performing a blocking study in an orthotopic mouse model. MM.1S_GFP+_/_Luc+_ cells were injected (IV) and allowed to grow for 15 days before a positive bioluminescence signal was observed in the spine and femur. For the blocking study, we pretreated a group of mice with unlabeled anti‐BCMA mAbs 24 h before IV injection (Figure S4, Supporting Information) and subsequently confirmed the specificity of the anti‐BCMA immuno‐nanoPET tracer to specifically target tumor cells in unblocked mice rather than to penetrate the tumor by passive internalization in both groups, which matched previously reported findings.^[^ ^14^ ^]^

**Figure 2:**
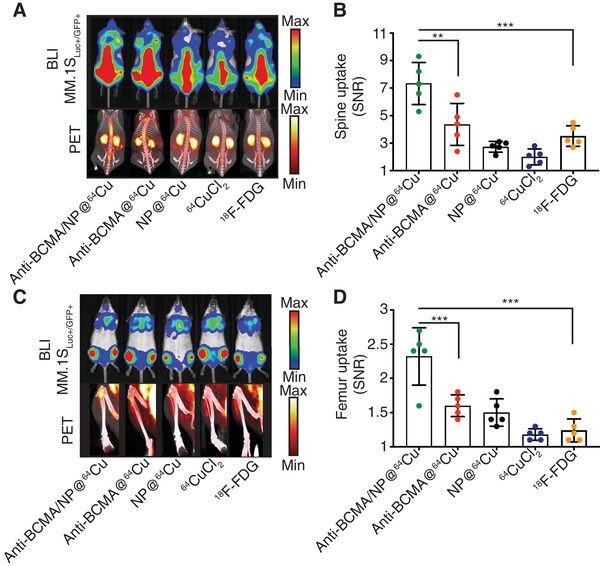
Anti‐BCMA immuno‐nanoPET radiotracers enhance the specificity of multiple myeloma detection. A) Bioluminescence imaging (BLI) of the dissemination of humanized myeloma cells (MM.1S) in SCID/beige mice confirmed the radiotracer presence in the spine of the animals. In parallel, mice were administered (IV) 10 MBq of radiotracers to evaluate their specificity to reach the tumor site located in the spine by whole‐body PET‐CT. Acquisitions were performed at *t* = 2 h for anti‐BCMA/NP@^64^Cu, *t* = 6 h for anti‐BCMA@^64^Cu, *t* = 15 min for NP@^64^Cu and ^64^CuCl_2,_ and *t* = 5 min for ^18^F‐FDG. B) Quantification of the signal‐to‐noise ratios (SNRs) was based on the PET signal emitted from the spine of the animals after imaging (*n* = 5/group). C) Qualitative imaging confirms the presence of tumor cells in the femurs by BLI and the uptake of the PET radiotracers. D) SNR quantification in the femurs of each mouse. All experiments were performed with *n* = 5 /group. The Mann‐Whitney test was performed for statistical analysis. **p < 0.01; ***p < 0.005.

To demonstrate the rationale of developing the anti‐BCMA PET nanoplatform, we next sought to compare the specificity of the anti‐BCMA immuno‐nanoPET tracer to conventional PET tracers in the same orthotopic model of MM (i.e., MM.1S_GFP+_/_Luc+_ cells implanted and allowed to grow for 15 days with positive bioluminescence signal confirmed in the spine and femur). We performed a quantification study based on equivalent quantities of radioisotope after IV administration (10 MBq). Each study group was imaged at their respective optimal time points: 5 min for ^18^F‐FDG, 15 min for ^64^CuCl_2_ and NP@^64^Cu, 2 h for anti‐BCMA/NP@^64^Cu, and 6 h for anti‐BCMA@^64^Cu (**Figure** **2** and Figure S5, Supporting Information). We observed a significantly brighter signal in the spines (Figure 2A,B) and femurs (Figure 2C,D) of mice administered with the anti‐BCMA/NP@^64^Cu radiotracer, which can be explained by the greater numbers of radiotracer in these organs (8.2 ± 2.1%ID/g and 1.3 ± 0.4%ID g^−1^, respectively) in comparison to the anti‐BCMA@^64^Cu immunoPET tracer (3.8 ± 1.4%ID g^−1^, *p* < 0.01 and 0.6 ± 0.2%ID g^−1^, *p* < 0.001, respectively) as well as the passive control groups, consisting of NP@^64^Cu (spine: 2.1 ± 0.3%ID g^−1^, *p* < 0.001; femur: 0.4 ± 0.4%ID g^−1^, *p* < 0.001) and the ^64^CuCl_2_ radiotracer.

Each agent that is currently used in clinical settings provided a very low signal in the two observed tumor sites (spine: 1.6 ± 0.2%ID g^−1^, *p* < 0.001; femur: 0.001 ± 2.001, *p* < 0.001). Signals from ^18^F‐FDG were mostly observed in the spine at significantly lower levels than the signals observed with the immuno‐nanoPET and immunoPET approaches. In addition, almost no signal was observed in the mouse femurs (Figure 2C,D). These results confirmed the need to design novel targeted PET imaging agents that are more specific than conventional ^18^F‐FDG and other untargeted radioisotopes. The lack of specificity of ^18^F‐FDG in these mice can be attributed to the low number of tumor cell clusters, which decreases the detection ability of ^18^F‐FDG and which is consistent with previous observations.^[^ ^14^ ^]^ The stronger signals in the spines and femurs of animals in the anti‐BCMA/NP@^64^Cu group when compared to those in the anti‐BCMA@^64^Cu group were attributed to the differences in the amounts of mAb injected (1.12 mg kg^−1^ vs 4.5 mg kg^−1^, respectively), which lead to lower off‐target effects and, hence, better overall uptake at the tumor sites.

**Figure 3:**
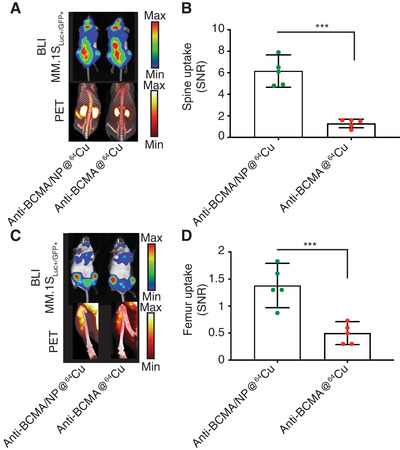
Anti‐BCMA immuno‐nanoPET radiotracers improve the sensitivity of multiple myeloma detection. A) Bioluminescence imaging (BLI) of the dissemination of humanized myeloma cells (MM.1S) in SCID/beige mice confirmed radiotracer presence in the spines of the animals. Anti‐BCMA/NP@^64^Cu and anti‐BCMA@^64^Cu were IV injected at a dose of 4.5 mg kg^−1^ mAb. B) Quantification of the PET radiotracers confirmed the improved sensitivity of the anti‐BCMA immuno‐nanoPET over anti‐BCMA immunoPET. C) Similarly, imaging of the femurs and D) quantitative analysis of the SNR demonstrated the improved sensitivity of anti‐BCMA/NP@^64^Cu to detect MM cells. All experiments were performed with *n* = 5/group. The Mann‐Whitney test was performed for statistical analysis. ***p < 0.005.

Finally, we sought to compare the sensitivity of anti‐BCMA/NP@^64^Cu to anti‐BCMA@^64^Cu. For this purpose, we injected groups of mice with the respective agents at the same doses based on anti‐BCMA mAb (4.5 mg kg^−1^) (**Figure** **3**). It appeared that immuno‐nanoPET tracers significantly improved the SNR compared to the anti‐BCMA immunoPET tracers. With a signal intensity increase of approximately five‐fold (*p* < 0.001, two‐way ANOVA) in the spine (Figure 3A,B) and approximately three‐fold in the femur (Figure 3C,D); the anti‐BCMA immuno‐nanoPET radiotracers allowed for improved sensitivity with potentially lower risks of antibody‐associated toxicity when compared to the immunoPET radiotracer. These improvements were attributed to the increased amount (12*x*) of radioisotope per mAb when comparing anti‐BCMA/NP@^64^Cu with anti‐BCMA@^64^Cu.

## 3 Conclusion

We demonstrate that ultrasmall targeted NPs may outperform conventional PET tracers for MM detection. Attributable to the superior amounts of PET tracer per mAb that may be achieved in the immune‐nanoPET platform, an anti‐BCMA/NP@^64^Cu radiotracer was developed and demonstrated to enable improvements in imaging sensitivity and specificity when compared with immunoPET and passively targeted PET radiotracers, including ^64^CuCl_2_, ^18^F‐FDG, and untargeted NPs@^64^Cu over the time course studies in this study. The use of anti‐BCMA/NP@^64^Cu allowed maximal contrast as quickly as 30 min after injection, potentially enabling its translation into a clinical workflow. In addition, its short half‐life in the body affords an optimal diagnostic tool that can be readministered frequently in the context of longitudinal monitoring. We observed rapid renal clearance of the complex after intravenous injection; but, the DLS measurements did not show any degradation of the complex in the urine of the animals, which is similar to previously reported results.^[^ ^14^ ^]^ Moreover, we did not observe higher uptake of this new agent into the liver than observed with the previous anti‐BCMA immunoPET tracers. While our results suggest significant improvements from our previous generations of anti‐BCMA ultrasmall NPs made with Gd^3+^ chelates for MRI‐based detection, it is important to note that further studies are necessary to understand the full elimination of antibody‐targeted ultrasmall immuno‐nanoPET tracers.

Finally, this targeted immuno‐nanoPET platform introduces potential clinical innovations that could circumvent the sensitivity limitations of analogous immuno‐MRI contrast agents. The enhanced sensitivity afforded for the detection of heterogeneous disease foci, as evidenced in the femurs of study mice, could promote clinical detection of disease missed by bone marrow biopsy or by am MR imaging technique that could be based on the previously designed anti‐BCMA targeted MRI contrast agent.^[^ ^14^ ^]^ Therefore, the current study could pave a path towards clinical translation of this innovative immuno‐nanoPET platform in MM patients and open the door for future therapeutic approaches where the radioisotope ^64^Cu may be replaced by ^67^Cu.

## 4 Experimental Section

### Synthesis of the Nanoparticles

Polysiloxane‐based NPs (obtained from NH TherAguix) were synthesized by previously described methods.^[^ ^23^ ^]^ Briefly, a gadolinium oxide (Gd_2_O_3_) core was synthesized in DEG (diethylene glycol) and encapsulated in a polysiloxane shell comprised of TEOS (tetraethyl orthosilicate) and APTES (amino‐propyltriethoxysilane). The surfaces of the NPs were functionalized by DOTAGA chelators (1,4,7,10‐tetra‐azacyclododecane‐1‐glutaric anhydride‐4,7,10‐triacetic acid) via the formation of a covalent amide bond between the primary amino group of APTES and the anhydride of the chelates. The nanoconstructs were then transferred from DEG to water, leading to Gd_2_O_3_ core dissolution and the complexation of Gd^3+^ ions by DOTAGA. Because of this dissolution, the polysiloxane hollow matrix collapsed and fragmented, leading to ultrasmall NPs that presented a hydrodynamic diameter less than 5 nm (3.6 ± 0.8 nm) and were composed of a polysiloxane core surrounded by approximately ten DOTAGA‐Gd^3+^ chelates. The number of free chelates (not complexing Gd^3+^) available at the surface of the NPs was found to be low (≈1%).^[^ ^22^ ^]^ Additionally, amino groups remained on the surface and could be used for further functionalization.

### Synthesis of the NODAGA‐Nanoparticles

NP@NODAGA was synthesized via the formation of a covalent amide bond between the abovementioned NPs, presenting free amino groups and NODAGA‐NHS chelates (2,2'‐(7‐(1‐carboxy‐4‐((2,5‐dioxopyrrolidin‐1‐yl)oxy)‐4‐oxobutyl)‐1,4,7‐triazonane‐1,4‐diyl)diacetic acid) as previously described.^[^ ^22^ ^]^ First, the NPs were incubated with NODAGA‐NHS in water at neutral pH and at RT for 5.5 h. They were then purified by tangential filtration over a 5 kDa cutoff membrane under acidic conditions to remove the nongrafted chelates. Purification by HPLC followed (Figure S1, Supporting Information). The obtained nanostructure presented a hydrodynamic diameter of less than 5 nm (4.3 ± 0.9 nm), which is known to be favorable for renal elimination. The addition of NODAGA at the particle surface was confirmed by the decrease in the isoelectric point (from 7.6 mV for NP to 5.2 mV for NP/NODAGA). This evolution is consistent with the exchange of the amino groups at the surface for carboxylic acid moieties. In addition, the infrared spectrum of NP/NODAGA showed the appearance of a vibration band at 1720 cm^−1^, corresponding to the elongation vibration of the C═O bonds of the carboxylic acids of NODAGA (Figure S1, Supporting Information). The numbers of NODAGA chelators added to the particle surface was quantified using two different techniques: titration with europium ions (Eu^3+^) as previously described^[^ ^22^ ^]^ and titration with copper ions (Cu^2+^) followed by HPLC (see Supporting Information). Both techniques gave consistent results of approximately 4 NODAGA chelators per particle. In addition, the determinations of the Gd, Si, C, and N content of the particles were carried out with ICP‐MS; and, the following formula was proposed: Gd_10_APTES*_33_TEOS*_41_DOTAGA*_10_._2_NODAGA*_4.1_ where APTES*, TEOS*, DOTAGA* and NODAGA* refer to the corresponding molecules that reacted and that are actually present within each NP. This conjugation method allowed an average of 4 NODAGA loaded per NP.

### Anti‐BCMA Radiolabeling

The anti‐BCMA immunoconjugates were radiolabeled with ^64^Cu, using a freeze‐dried kit based on an established protocol^[^ ^24^ ^]^ that contains 4 mg of anti‐BCMA monoclonal antibody (mAb) (BioLegend Inc. San Diego, CA, USA – Cat. No. 357502). DOTA‐NHS‐ester was purchased from Macrocyclics (Plano, Tx, USA). Briefly, 10 mg ml^−1^ anti‐BCMA mAbs were incubated with DTPA (40 mM; 4 °C for 30 min), loaded on a PD‐10 column (Sephadex G25, GE Healthcare) and eluted with phosphate buffer (0.05 M; pH 7.0). The concentration of mAb was determined by the Bradford assay prior to mAb incubation with a 100‐fold molar excess of DOTA‐NHS‐ester (4 °C for 24 h). The reaction mixture was transferred to an ammonium acetate buffer (0.5 M; pH 5.5) to remove excess unbound DOTA. The final concentration of mAb was assessed by the Bradford assay. For the radiolabeling process, mAbs in ammonium acetate buffer (0.3 M; pH 7.0) were incubated with 500 MBq of ^64^CuCl_2_ dissolved in a 250 µl solution of HCl (0.1 N; 1.5 h and at 40 °C). The labeling purity was assessed by instant thin layer chromatography (iTLC‐SG) in citrate buffer (pH 4.5; 0.1 M) and was determined to be 86%.

NODAGA‐NPs were conjugated to the mAbs by using a previously reported transcyclooctene‐tetrazin click chemistry approach.^[^ ^21^ ^]^ Briefly, NPs@NODAGA (50 mM) was mixed in a 1:10 molar ratio with NHS‐PEG4‐Tz linker for 30 min at RT. Non‐bound NHS‐Tz was removed by tangential ultracentrifugation, using a Vivaspin device (MWCO = 3 kDa). In parallel, anti‐BCMA mAb was co‐incubated with NHS‐PEG4‐TCO linkers at RT for 3 h followed by centrifugation filtration (15 000 rcf), using a 50 kDa molecular weight cutoff membrane (Millipore), before resuspension in PBS. This process was performed in triplicate to remove all unbound NPs. These surface‐modified NPs were subsequently mixed at a 100:1 molar ratio with the anti‐BCMA mAb and stirred at RT for 1 h. Centrifugation filtration (15 000 rcf) was performed by using a 50 kDa molecular weight cutoff membrane (Millipore) before resuspension in PBS. This process was performed in triplicate to remove all unbound NPs. Labeling of NP@^64^Cu and anti‐BCMA/NP@^64^Cu was performed by using the same protocol as employed for labeling of the immunoconjugate mAb. The final concentration of NPs was assessed by ICP‐MS; and, the final concentration of mAb was assessed by the Bradford assay. We observed an average of 3 NPs per mAb, and hence an average of 12 NODAGA chelators per mAb for the anti‐BCM/NP constructs.

### Cell Lines

The MM.1S cell line was purchased from ATCC (Manassas, VA, USA). Cells were transduced using the pGC‐GFP/Luc vector and authenticated by short tandem repeat DNA profiling. MM.1S cells were cultivated in RPMI medium containing 10% fetal bovine serum, 1% penicillin/streptavidin, and 1% glutamine.

### Animals

All mouse work was performed in accordance with the IACUC of the Dana‐Farber Cancer Institute (protocol 14‐001). A total of 5 × 10^6^ MM.1S_GFP+/Luc+_ cells were injected (IV) into SCID/beige mice (*n* = 5/group) for dissemination in the bone marrow and femurs, establishing the orthotopic xenograft MM mouse model. The same 5 mice/group underwent the bioluminescence and PET‐CT imaging study.

### Bioluminescence Imaging (BLI)

To monitor tumor dissemination, mice were injected (I.P.) with luciferin (100 µl) and placed under anesthesia with 5% isoflurane. Animals were then randomly assigned to each study group and tumor dissemination was tracked by the BLI signal, using the IVIS system (Perkin Elmer).

### PET‐CT imaging

PET‐CT scans were obtained on a Inveon multimodality system (Siemens Medical Solutions USA Inc.). The CT scans were performed using low‐dose radiation based on preliminarily established protocols^[^ ^14^, ^21^ ^]^ (80 kVp, 0.5 mA, 220degree rotation, 600 ms per degree exposure time, 80 µm reconstruction pixel time) for both anatomical reference and delineation of the region and volume of interest (ROI/VOI). PET acquisitions were performed after IV injection of the radiotracers. For ^18^F‐FDG imaging, mice that were fastered for 12 h prior to image acquisition, anesthetized with 5% isoflurane, and administered (IV) 10 MBq of radiotracer (200 µl). For ^64^CuCl_2_ acquisitions, similar procedures were performed but without requisite animal fasting. Similarly, PET imaging with anti‐BCMA@^64^Cu, NP@^64^Cu, and anti‐BCMA/NP@^64^Cu was performed after injection (IV) based on equivalent amounts of radiotracer (10 MBq) for specificity comparisons or of antibody (4.5 mg kg^−1^) for sensitivity comparisons. The VOIs were drawn manually by using the Inveon Research Workplace software and were converted to the percentage of the injected radioactive dose per g of tissue (%ID g^−1^) by assuming a 1 g ml^−1^ tissue density. Correlations of the radiation decay were performed to compare biodistribution data. The SNR was calculated as the intensity/noise ratio, where the noise was the signal intensity emitted in the muscle of the analyzed mouse.

### Biodistribution and Pharmacokinetic Study

A total of *n* = 5/time point/group (different from the imaging studies) were used for biodistribution and pharmacokinetic studies. Blood and major organs, including bone marrow (from the spine and femurs), liver, kidney, lung, muscle, spleen, brain, and heart, were dissected, weighed, and counted on a calibrated and normalized gamma counter.

### Statistical Analysis

Statistical analyses were performed using GraphPad Prism software V.9.0. Sensitivity and specificity differences were assessed by the Mann‐Whitney test. A *p‐*value < 0.05 was considered significant.

## Conflict of Interest

F.L. and O.T., who are inventors of WO2011/135 101 that protects the based NPs described in this publication, are employees of NH TherAguix. F.L., O.T., A.D. are shareholders of NH TherAguix, which is developing the base NP technology for commercial purposes.

## Supporting information

Supporting Information

## Data Availability

Research data are not shared.

## References

[1] C. P. Shortt , T. G. Gleeson , K. A. Breen , J. McHugh , M. J. O'Connell , P. J. O'Gorman , S. J. Eustace , AJR Am. J. Roentgenol. 2009, 192, 980. 19304704 10.2214/AJR.08.1633

[2] A. G. Ormond Filho , B. C. Carneiro , D. Pastore , I. P. Silva , S. R. Yamashita , F. D. Consolo , V. T. M. Hungria , A. F. Sandes , E. G. Rizzatti , M. A. C. Nico , Radiographics 2019, 39, 1077. 31283452 10.1148/rg.2019180096

[3] C. Nanni , Cancers 2020, 12, 1030. 32331374 10.3390/cancers12041030 PMC7226158

[4] S. Mule , E. Reizine , P. Blanc‐Durand , L. Baranes , P. Zerbib , R. Burns , R. Nouri , E. Itti , A. Luciani , Cancers 2020, 12, 3155. 33121132 10.3390/cancers12113155 PMC7693006

[5] H. Maeda , J. Wu , T. Sawa , Y. Matsumura , K. Hori , J. Controlled Release 2000, 65, 271. 10.1016/s0168-3659(99)00248-5 10699287

[6] Y. Shou , J. Lu , T. Chen , D. Ma , L. Tong , J. Cancer Res. Ther. 2012, 8, 96. 22531522 10.4103/0973-1482.95182

[7] J. R. Bading , A. F. Shields , J. Nucl. Med. 2008, 49, 64S. 18523066 10.2967/jnumed.107.046391

[8] Y. Abe , S. Ikeda , A. Kitadate , K. Narita , H. Kobayashi , D. Miura , M. Takeuchi , E. O'Uchi , T. O'Uchi , K. Matsue , Eur. J. Nucl. Med. Mol. Imaging 2019, 46, 1345. 30903198 10.1007/s00259-019-04312-9

[9] W. Wei , Z. T. Rosenkrans , J. Liu , G. Huang , Q. Y. Luo , W. Cai , Chem. Rev. 2020, 120, 3787. 32202104 10.1021/acs.chemrev.9b00738 PMC7265988

[10] C. Bailly , S. Gouard , F. Guerard , B. Chalopin , T. Carlier , A. Faivre‐Chauvet , P. Remaud‐Le Saec , M. Bourgeois , N. Chouin , L. Rbah‐Vidal , R. Tripier , F. Haddad , F. Kraeber‐Bodere , C. Bodet‐Milin , M. Cherel , Int. J. Mol. Sci. 2019, 20, 2564. 31137758 10.3390/ijms20102564 PMC6567828

[11] C. Bailly , S. Gouard , M. Lacombe , P. Remaud‐Le Saec , B. Chalopin , M. Bourgeois , N. Chouin , R. Tripier , Z. Halime , F. Haddad , A. Faivre‐Chauvet , F. Kraeber‐Bodere , M. Cherel , C. Bodet‐Milin , Oncotargets 2018, 9, 9061. 10.18632/oncotarget.23886 PMC5823645 29507674

[12] G. A. Ulaner , N. B. Sobol , J. A. O'Donoghue , A. S. Kirov , C. C. Riedl , R. Min , E. Smith , L. M. Carter , S. K. Lyashchenko , J. S. Lewis , C. O. Landgren , Radiology 2020, 295, 606. 32255416 10.1148/radiol.2020192621 PMC7263286

[13] C. Wang , Y. Chen , Y. N. Hou , Q. Liu , D. Zhang , H. Zhao , Y. Zhang , S. An , L. Li , J. Hou , G. Huang , J. Liu , Y. J. Zhao , W. Wei , Eur. J. Nucl. Med. Mol. Imaging 2021, 48, 2749. 33543326 10.1007/s00259-021-05218-1

[14] A. Detappe , M. Reidy , Y. Yu , C. Mathieu , H. V. Nguyen , T. P. Coroller , F. Lam , P. Jarolim , P. Harvey , A. Protti , Q. D. Nguyen , J. A. Johnson , Y. Cremillieux , O. Tillement , I. M. Ghobrial , P. P. Ghoroghchian , Nanoscale 2019, 11, 20485. 31650133 10.1039/c9nr06512a

[15] N. Raje , J. Berdeja , Y. Lin , D. Siegel , S. Jagannath , D. Madduri , M. Liedtke , J. Rosenblatt , M. V. Maus , A. Turka , L. P. Lam , R. A. Morgan , K. Friedman , M. Massaro , J. Wang , G. Russotti , Z. Yang , T. Campbell , K. Hege , F. Petrocca , M. T. Quigley , N. Munshi , J. N. Kochenderfer , N. Engl. J. Med. 2019, 380, 1726. 31042825 10.1056/NEJMoa1817226 PMC8202968

[16] S. Hipp , Y. T. Tai , D. Blanset , P. Deegen , J. Wahl , O. Thomas , B. Rattel , P. J. Adam , K. C. Anderson , M. Friedrich , Leukemia 2017, 31, 1743. 28025583 10.1038/leu.2016.388

[17] S. F. Cho , L. Lin , L. Xing , Y. Li , T. Yu , K. C. Anderson , Y. T. Tai , Cancers 2020, 12, 1473. 32516895 10.3390/cancers12061473 PMC7352710

[18] A. Detappe , S. Kunjachan , P. Drane , S. Kotb , M. Myronakis , D. E. Biancur , T. Ireland , M. Wagar , F. Lux , O. Tillement , R. Berbeco , Sci. Rep. 2016, 6, 34040. 27658637 10.1038/srep34040 PMC5034311

[19] A. Detappe , S. Kunjachan , L. Sancey , V. Motto‐Ros , D. Biancur , P. Drane , R. Guieze , G. M. Makrigiorgos , O. Tillement , R. Langer , R. Berbeco , J. Controlled Release 2016, 238, 103. 10.1016/j.jconrel.2016.07.021 27423325

[20] A. Detappe , E. Thomas , M. W. Tibbitt , S. Kunjachan , O. Zavidij , N. Parnandi , E. Reznichenko , F. Lux , O. Tillement , R. Berbeco , Nano Lett. 2017, 17, 1733. 28145723 10.1021/acs.nanolett.6b05055 PMC5505266

[21] A. Detappe , C. Mathieu , C. Jin , M. P. Agius , M. C. Diringer , V. L. Tran , X. Pivot , F. Lux , O. Tillement , D. Kufe , P. P. Ghoroghchian , Int. J. Radiat. Oncol., Biol., Phys. 2020, 108, 1380. 32634545 10.1016/j.ijrobp.2020.06.069 PMC7680267

[22] P. Bouziotis , D. Stellas , E. Thomas , C. Truillet , C. Tsoukalas , F. Lux , T. Tsotakos , S. Xanthopoulos , M. Paravatou‐Petsotas , A. Gaitanis , L. A. Moulopoulos , V. Koutoulidis , C. D. Anagnostopoulos , O. Tillement , Nanomedicine 2017, 12, 1561. 28621567 10.2217/nnm-2017-0032

[23] G. L.e Duc , S. Roux , A. Paruta‐Tuarez , S. Dufort , E. Brauer , A. Marais , C. Truillet , L. Sancey , P. Perriat , F. Lux , O. Tillement , Cancer Nanotechnol. 2014, 5, 4. 26561512 10.1186/s12645-014-0004-8 PMC4631720

[24] W. Wojdowska , U. Karczmarczyk , M. Maurin , P. Garnuszek , R. Mikolajczak , Curr. Radiopharm. 2015, 8, 62. 25506704 10.2174/1874471008666141215151253

