# Supplementary material for: Anti‐BCMA Immuno‐NanoPET Radiotracers for Improved Detection of Multiple Myeloma
Source: Adv Healthc Mater. 2021 Nov 7;11(2):2101565. doi: 10.1002/adhm.202101565 (PMC11469021; doi:10.1002/adhm.202101565)
Supplement: Supplementary file 1 — Supporting Information [file ADHM-11-2101565-s001.pdf]

© 2021 Wiley-VCH GmbH

ADVANCED HEALTHCARE MATERIALS

Supporting Information

for *Adv. Healthcare Mater.*, DOI: 10.1002/adhm.202101565

Anti-BCMA Immuno-nanoPET Radiotracers for Improved Detection of Multiple Myeloma

Eloise Thomas[1], Clélia Mathieu[2], Patricia Moreno-Gaona[3], Vincent Mittelheisser[3], François Lux[4,5], Olivier Tillement[4], Xavier Pivot[3], P. Peter Ghoroghchian[6,7,*], and Alexandre Detappe[3,8,*]

SUPPLEMENTATY INFORMATION

Anti-BCMA Immuno-nanoPET Radiotracers for Improved Detection of Multiple Myeloma

Eloise Thomas[1], Clélia Mathieu[2], Patricia Moreno-Gaona[3], Vincent Mittelheisser[3], François Lux[4,5], Olivier Tillement[4], Xavier Pivot[3], P. Peter Ghoroghchian[6,7,*], and Alexandre Detappe[3,8,*]

1. LAGEPP Université Claude Bernard Lyon 1, CNRS UMR5007, Villeurbanne, France France
2. Université Paris-Saclay, CNRS UMR 8612, Institut Galien Paris-Saclay, France
3. Institut de Cancérologie Strasbourg Europe (ICANS), Strasbourg, France
4. Institut Lumière-Matière, Université Claude Bernard Lyon 1, CNRS UMR5306, Villeurbanne, France.
5. Institut Universitaire de France (IUF), Paris, France
6. David H Koch Institute for Integrative Cancer Research, MIT, Cambridge MA, USA
7. Dana Farber Cancer Institute, Boston MA, USA
8. Strasbourg Drug Discovery and Development Institute (IMS), Strasbourg, France

*Corresponding authors: P Peter Ghoroghchian, MD, PhD ppg@mit.edu; Alexandre Detappe, PhD: a.detappe@icans.eu

SUPPLEMENTARY MATERIAL AND METHODS

Synthesis and characterizations of NP/NODAGA

The functionalization of ultrasmall polysiloxane-based nanoparticles (NP)[1,2] with NODAGA chelators was performed following a previously published report[3]. The purification was performed by tangential filtration over a 5 kDa cut-off membrane in acidic conditions. The removal of non-grafted NODAGA was followed by titration with an excess of Cu^{2+} followed by HPLC, *vide infra*, to resolved Cu^{2+}, to non-grafted NODAGA@Cu^{2+} and to NP/NODAGA@Cu^{2+}. After purification, NP/NODAGA were characterized with several complementary techniques as described below and as summarized in **Supplementary Table 1** and **Supplementary Figure 1**.

Chemicals: Sodium hydroxide (NaOH, 99.99%) and hydrochloric acid (HCl, 36.5−38%) were purchased from Aldrich Chemical (France. Acetonitrile (CH3CN, >99.9%) was purchased from Carlo Erba (France). Trifluoroacetic acid (TFA, >99%) was purchased from Alfa Aesar (United Kingdom). Copper sulfate pentahydrate (CuSO4.5H2O, 98%) was purchased from Merck (Germany). The NODAGA chelate (2,2'-(7-(1-carboxy-4-((2,5-dioxopyrrolidin-1-yl)oxy)-4-oxobutyl)-1,4,7-triazonane-1,4-diyl)diacetic acid) was purchased from ChemaTech (Dijon, France). All products were used without further purification. Only Mili-Q water (ρ>18MΩ.cm) was used for aqueous solution preparation.

Purification monitoring and quantification of NODAGA available at the particle's surface with Cu^{2+} titration followed by HPLC: NODAGA chelator in the presence of copper Cu^{2+} form a complex (logK = 21.6 for NOTA@Cu^{2+} [4]) that absorsbs strongly at 700 nm and allows them to be detected easily. To follow the purification of nanoparticles and to quantify the amounts of NODAGA at the particle surface, an excess of Cu^{2+} was added followed by HPLC analysis to resolved Cu^{2+}, to non-grafted NODAGA@Cu^{2+} and to NP/NODAGA@Cu^{2+} (**Supplementary Figure S1**). An excess of Cu^{2+} was added to a solution of NP/NODAGA at a concentration in gadolinium higher than 50 mM. The complexation of Cu^{2+} was carried out for a pH of about 3 in order to avoid the presence of copper hydroxide (pKs = 19.3 [5]). Solutions were then heated in an oven at 80°C for 2 h. Then solutions were injected into HPLC to separate free Cu^{2+}, NODAGA@Cu^{2+} and NP/NODAGA@Cu^{2+}. Comparison with calibration curve of NODAGA/Cu^{2+} lead to the quantification of NODAGA added at the surface of the nanoparticle (about 4.3 NODAGA per particles).

HPLC characterizations: Gradient HPLC analysis was done by using a Shimadzu® Prominence series UFLC system with a CBM-20A controller bus module, an LC-20AD liquid chromatograph, a CTO-20A column oven, an SPD-20A UV-visible detector and an RF-20A fluorescence detector. UV-visible absorption was measured at 700 nm (UV-visible absorption single wavelength detection). 20 µL of the sample were loaded in the solvent injection ratio: 99 % solvent A–1 % solvent C (A = Milli-Q water–TFA 99.9 : 0.1 v/v; C = CH3CN–TFA 99 : 9.1 : 0.1 v/v/v) onto a Jupiter C4 column (150 × 4.60 mm, 5 µm, 300 Å, Phenomenex®) at a flow rate of 1 $mL.min^{-1}$ for 7 min. In a second step, samples were eluted

by a gradient developed from 1 to 90% of solvent C for 15 min. The concentration of solvent C was maintained for 7 min. Then, the concentration of solvent C was decreased to 1% for a period of 1 min followed by an additional 8 min at this final concentration to re-equilibrate the system. Before each sample measurement, a baseline was performed following the same conditions by loading Milli-Q water into the injection loop. Free Cu^{2+} had a retention time of 2.5 min; NODAGA@Cu^{2+} had a retention time of 3 min; and, NP/NODAGA@Cu^{2+} had a retention time of 14 min (**Supplementary Figure S1**).

Infrared Spectroscopy: Solutions of NP and NP/NODAGA were acidified before evaporating the water in the oven. Infrared spectra were obtained with an IRAffinity-1 Shimadzu instrument with an ATR-FTIR (Attenuated Total Reflection Fourrier Transform Infrared) platform in the range of 550 to 4000 cm^{-1}. The IR spectra obtained on the recovered powders are presented in **Supplementary Figure S1**. In contrast to the NP spectra, the NP/NODAGA spectra shows a band at 1720 cm^{-1} which corresponds to the elongation vibration of the C=O bonds of the carboxylic acids of the NODAGA [6,7].

Size, zeta potential: these parameters were determined as described by Bouziotis *et al.*[3]. Hydrodynamic diameter was found to be 3.6 ± 0.8 nm for NP and 4.3 ± 0.9 nm for NP/NODAGA. The isoelectric point was found to be 7.6 mV for NP and 5.2 mV for NP/NODAGA.

Chemical analysis: The determination of the Gd, Si, C, and N content of particles was performed by the Filab (France) by ICP-MS (precision: 0.4%). For NP/NODAGA, mass percentages of 8.5 for Gd, 11.5 for Si, 23.31 for C and 6.44 for N were determined. These results were consistent with the following average composition for NP/NODAGA: ($Gd_1APTES*_{3.3}TEOS*_{4.1}DOTAGA*_{1.02}NODAGA*_{0.41}$). x H2O. APTES*, TEOS*, DOTAGA* and NODAGA* refer to the corresponding molecules that have reacted and found to be present within the NPs. This formula gives 1 NODAGA* for 2.4 Gd^{3+} which mean about 4.2 NODAGA per particle.

Quantification of NODAGA available at the particle's surface with Eu^{3+}: In order to quantify more precisely the numbers of NODAGA grafted on the NP surface, we performed a titration based on europium (Eu) luminescence[3]. This led to a determined value of 4.1 NODAGA per particle.

Supplementary Table 1: Nanoparticles characterizations before and after functionalization with NODAGA.

**Characterization**	**NP**	**NP/NODAGA**
Size	3.6 ± 0.8 nm	4.3 ± 0.9 nm
Isoelectric point	7.6 mV	5.2 mV
Infrared spectra	No band at 1720 cm^{-1}	Band at 1720 cm^{-1} (carboxylic acid from NODAGA)
Mass percentage (%) from chemical analysis	14.0 for Gd, 11.5 for Si, 29.0 for C and 8.1 for N	8.5 for Gd, 11.5 for Si, 23.3 for C and 6.4 for N
Proposed composition from chemical analysis [(a)]	$Gd_{10}APTES*_{25}TEOS*_{21}$ $DOTAGA*_{10}$	$Gd_{10}APTES*_{33}TEOS*_{41}$ $DOTAGA*_{10}NODAGA*_{4}$.
Number of NODAGA [(b)]	x	~4 per particle

[(a)] *APTES*, TEOS*, DOTAGA* and NODAGA* refer to the corresponding molecules that have reacted and found to be present within the nanoparticle.*

[(b)] *The numbers of NODAGA per particle as determined by the following 3 techniques were found to be consistent: titration with Eu^{3+}, titration with Cu^{2+} followed by HPLC, and chemical analysis.*

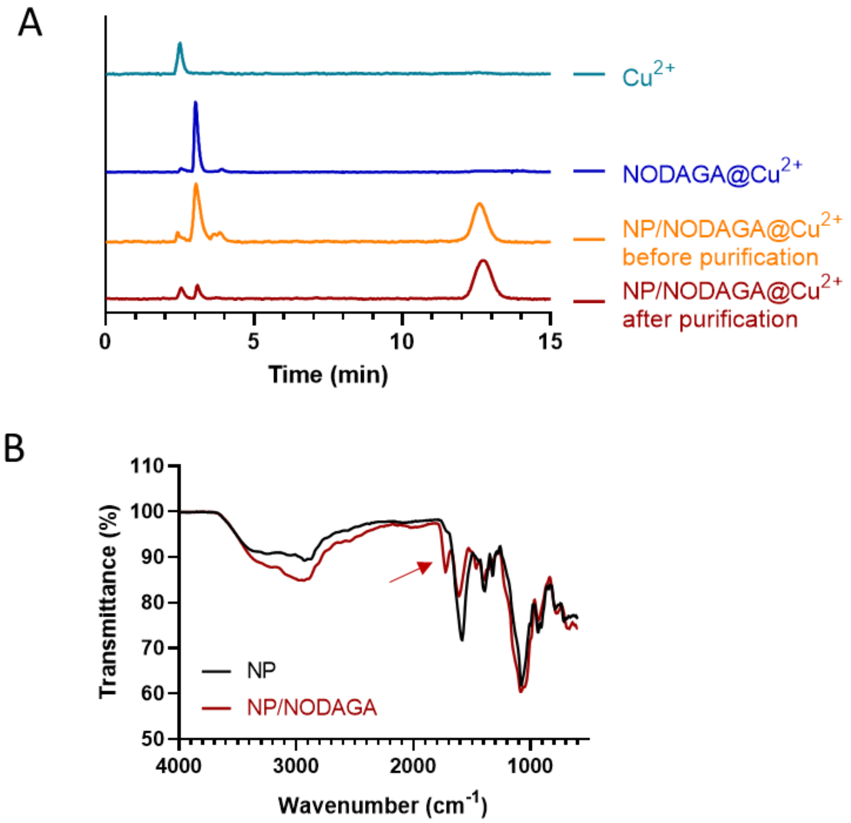

Supplementary Figure S1: **Synthesis and characterization of NP/NODAGA.** **A)** HPLC chromatograms of Cu^{2+}, NODAGA@Cu^{2+}, NP/NODAGA@Cu^{2+} before purification and NP/NODAGA@Cu^{2+} after purification. The almost complete disappearance of the peak at 3 min confirms the removal of non-grafted NODAGA. **B)** Infrared spectra of NP and NP/NODAGA. The appearance of the band at 1720 cm^{-1} confirms the grafting of NODAGA at the surface of the particle. 1720 cm^{-1}: elongation of the C=O of carboxylic acids; 1600 cm^{-1}: asymmetric elongation of the C=O of carboxylates, C=O of amides and deformation of the N-H of the amide; 1440 cm^{-1}: deformation of carboxylic acids; 1390 cm^{-1}: symmetric elongation of the C=O of carboxylates; 1080 cm^{-1}: asymmetric elongation of the Si-O-Si; 930 cm^{-1}: elongation of the Si-O [6,7].

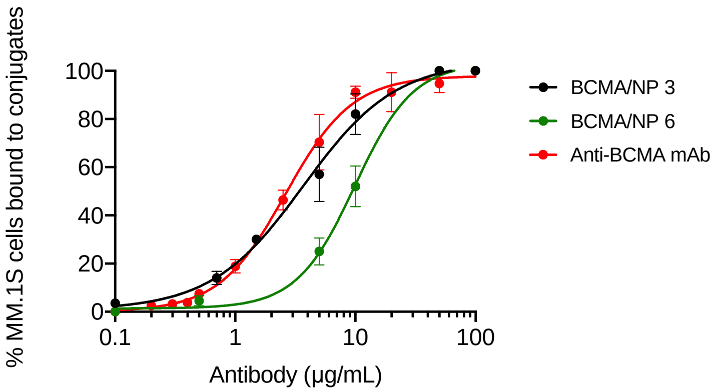

Supplementary Figure S2: Binding study performed by flow cytometry on MM.1S$_{GFP^+Luc^+}$ cells after 10 min of incubation at 4C with APC-labelled anti-BCMA mAb, and anti-BCMA mAbs conjugated to an average of 3 NPs or an average of 6 NPs.

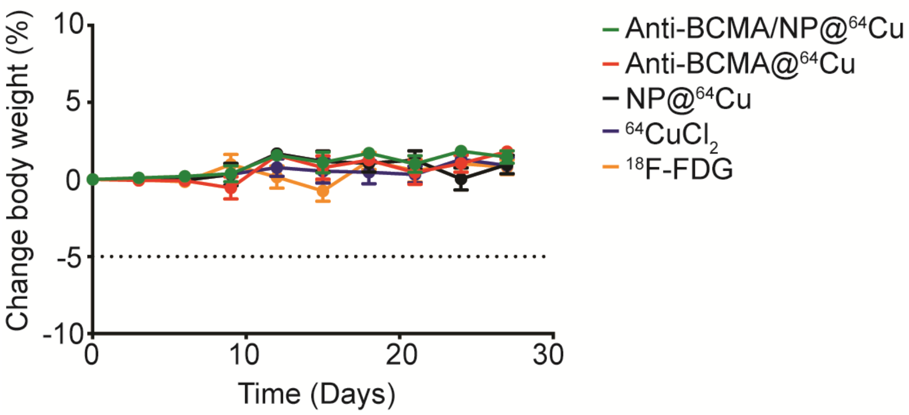

Supplementary Figure S3. Body weight measurements after single dose administration of various PET tracers at an equivalent dose level (10 MBq) based on radioisotope in healthy balb/c mice.

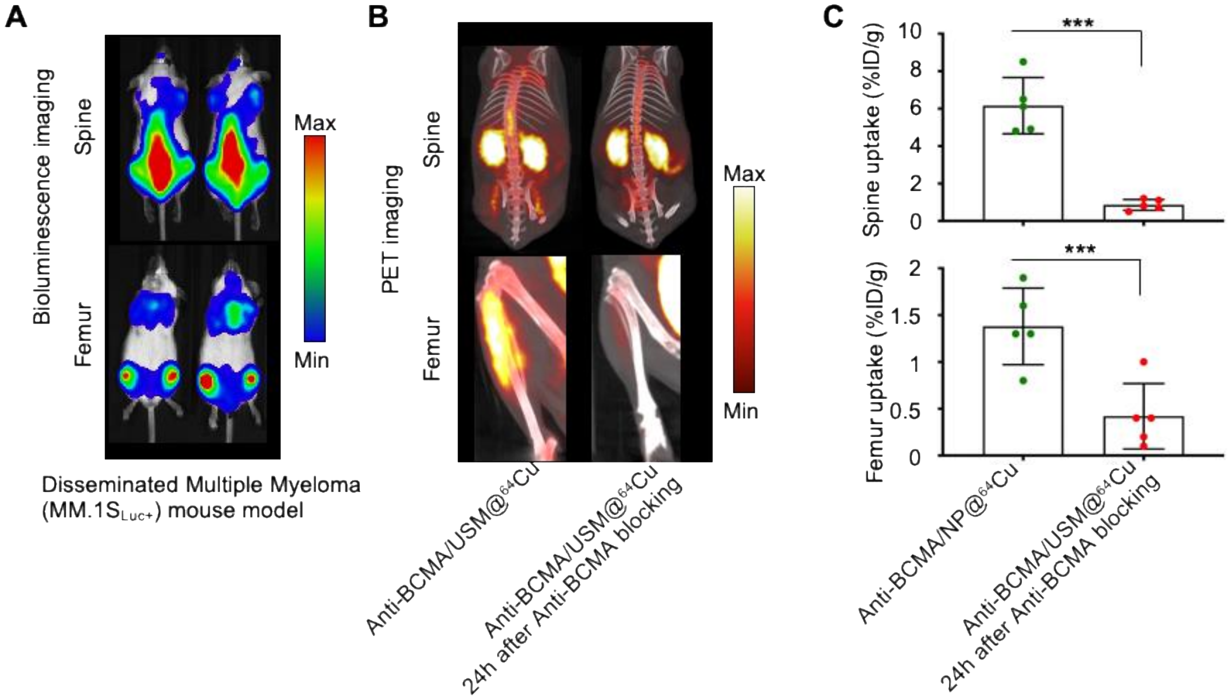

Supplementary Figure S4. In vivo BCMA targeting specificity study. Orthotopic xenograft MM.1S SCID-beige mice were pre-treated with anti-BCMA mAb (4.2 mg/kg for 24 h) before administration (IV) of the anti-BCMA/NP@^{64}Cu radiotracer. **A)** Bioluminescence imaging (BLI) confirms the presence of MM.1S cells in the spine; and, PET/CT imaging at 30 min after injection of anti-BCMA/NP@64Cu demonstrates a hyperfixation spot in the spine for the untreated group (n=5/group). **B)** Quantification of the amount of ^{64}Cu in the spine confirms the specificity of the anti-BCMA/NP@^{64}Cu to target MM.1S cells *in vivo*. **C)** Similarly, qualitative comparison in the femurs and **D)** quantification of the amount of 64Cu in the femurs confirms the specificity of the anti-BCMA/NP@64Cu to target MM.1S cells.

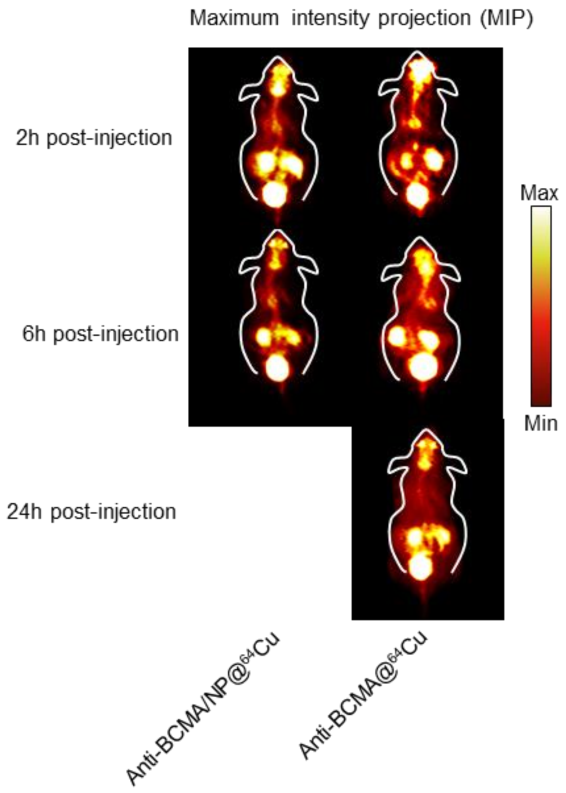

Supplementary Figure S5: Maximum intensity projections of the mice injected with anti-BCMA/NP@^{64}Cu and Anti-BCMA@^{64}Cu

References

[1] A. Detappe, S. Kunjachan, L. Sancey, V. Motto-Ros, D. Biancur, P. Drane, R. Guieze, G. M. Makrigiorgos, O. Tillement, R. Langer, R. Berbeco. *J Control Release* **2016**, *238*, 103.

[2] F. Lux, V. L. Tran, E. Thomas, S. Dufort, F. Rossetti, M. Martini, C. Truillet, T. Doussineau, G. Bort, F. Denat, F. Boschetti, G. Angelovski, A. Detappe, Y. Cremillieux, N. Mignet, B. T. Doan, B. Larrat, S. Meriaux, E. Barbier, S. Roux, P. Fries, A. Muller, M. C. Abadjian, C. Anderson, E. Canet-Soulas, P. Bouziotis, M. Barberi-Heyob, C. Frochot, C. Verry, J. Balosso, M. Evans, J. Sidi-Boumedine, M. Janier, K. Butterworth, S. McMahon, K. Prise, M. T. Aloy, D. Ardail, C. Rodriguez-Lafrasse, E. Porcel, S. Lacombe, R. Berbeco, A. Allouch, J. L. Perfettini, C. Chargari, E. Deutsch, G. Le Duc, O. Tillement. *Br J Radiol* **2019**, *92*, 20180365.

[3] P. Bouziotis, D. Stellas, E. Thomas, C. Truillet, C. Tsoukalas, F. Lux, T. Tsotakos, S. Xanthopoulos, M. Paravatou-Petsotas, A. Gaitanis, L. A. Moulopoulos, V. Koutoulidis, C. D. Anagnostopoulos, O. Tillement. *Nanomedicine (Lond)* **2017**, *12*, 1561.

[4] G. Anderegg, F. Arnaud-Neu, R. Delgado, J. Felcman, K. Popov. *Pure and Applied Chemistry* **2005**, *77*, 1445.

[5] E. Protopopoff, P. Marcus. *Electrochimica Acta* **2005**, *51*, 408.

[6] B. L. Frey, R. M. Corn. *Analytical Chemistry* **1996**, *68*, 3187.

[7] M. Stoia, O. Ştefãnescu, G. Vlase, L. Barbu-Tudoran, M. Barbu, M. Ştefãnescu. *Journal of Sol-Gel Science and Technology* **2012**, *62*, 31.
